# Predicting biochemical recurrence of prostate cancer with artificial intelligence

**DOI:** 10.1038/s43856-022-00126-3

**Published:** 2022-06-08

**Authors:** Hans Pinckaers, Jolique van Ipenburg, Jonathan Melamed, Angelo De Marzo, Elizabeth A. Platz, Bram van Ginneken, Jeroen van der Laak, Geert Litjens

**Affiliations:** 1 Department of Pathology, Radboud Institute for Health Sciences, Radboud University Medical Center, Nijmegen, The Netherlands; 2 Department of Pathology, New York University Langone Medical Center, New York, NY USA; 3 Departments of Pathology, Urology and Oncology, The Brady Urological Research Institute and the Sidney Kimmel Comprehensive Cancer Center at Johns Hopkins, Baltimore, MD USA; 4 Department of Epidemiology, Johns Hopkins Bloomberg School of Public Health, Baltimore, MD USA; 5 Center for Medical Image Science and Visualization, Linköping University, Linköping, Sweden

**Keywords:** Prostate cancer, Prostate, Prognostic markers, Epidemiology

## Abstract

**Background:**

The first sign of metastatic prostate cancer after radical prostatectomy is rising PSA levels in the blood, termed biochemical recurrence. The prediction of recurrence relies mainly on the morphological assessment of prostate cancer using the Gleason grading system. However, in this system, within-grade morphological patterns and subtle histopathological features are currently omitted, leaving a significant amount of prognostic potential unexplored.

**Methods:**

To discover additional prognostic information using artificial intelligence, we trained a deep learning system to predict biochemical recurrence from tissue in H&E-stained microarray cores directly. We developed a morphological biomarker using convolutional neural networks leveraging a nested case-control study of 685 patients and validated on an independent cohort of 204 patients. We use concept-based explainability methods to interpret the learned tissue patterns.

**Results:**

The biomarker provides a strong correlation with biochemical recurrence in two sets (*n* = 182 and *n* = 204) from separate institutions. Concept-based explanations provided tissue patterns interpretable by pathologists.

**Conclusions:**

These results show that the model finds predictive power in the tissue beyond the morphological ISUP grading.

## Plain language summary

To determine the prognosis of patients with prostate cancer, several clinical factors are taken into account. One of these is the cancer grade, assigned by a pathologist based on the cancer’s appearance under a microscope. The grade ranges from 1 to 5, where 5 is the most aggressive tumour type. This study explored whether deep learning—a technique in which computer software learns patterns from multiple examples—can learn to predict the risk of patients’ cancers recurring from microscopic images of the tumours. We show, on two clinical datasets from different institutions, that such a system can help to better predict prognosis, beyond the information provided by grade alone. In the future, this type of method could help clinicians to predict the prognosis of individual prostate cancer patients.

## Abstract (web summary)

Pinckaers et al. develop a deep learning system to predict biochemical recurrence in prostate cancer patients treated with radical prostatectomy. The authors’ morphological biomarker provides predictive power beyond traditional Gleason grading, based on analysis of two clinical datasets from different institutions.

## Introduction

Prostate cancer is a common malignancy among men, affecting 1.4 million per year^1^. A significant proportion of these men will receive the primary curative treatment of a prostatectomy. This surgery’s success can partly be judged by the concentration of prostate-specific antigen (PSA) in the blood. While it has a dubious role in prostate cancer screening^2,3^, this protein is a valuable biomarker in PCa patients’ follow-up post-prostatectomy. In a successful surgery, the concentration will mostly be undetectable (<0.1 ng/mL) after 4–6 weeks^4^.

However, in ~30% of the patients^5–7^, PSA will rise again after surgery, called biochemical recurrence, pointing to regrowth of prostate cancer cells. Biochemical recurrence is a prognostic indicator for subsequent progression to clinical metastases and prostate cancer death^8^. Estimating chances of biochemical recurrence could help to better stratify patients for specific adjuvant treatments.

The risk of biochemical recurrence of prostate cancer is currently assessed in clinical practice through a combination of the ISUP grade^9^, the PSA value at diagnosis and the TNM staging criteria. In a recent European consensus guideline, these factors were proposed to separate the patients into a low-risk, intermediate-risk and high-risk group^10^. A high ISUP grade independently can, independently of other factors, assign a patient to the intermediate (grade 2/3) or high-risk group (grade 4/5).

Based on the distribution of the Gleason growth patterns^11^, which are prognostically predictive morphological patterns of prostate cancer, pathologists assign cancerous tissue obtained via biopsy or prostatectomy into one of five groups. They are commonly referred to as International Society of Urological Pathology (ISUP) grade groups, the ISUP grade, Gleason grade groups, or just grade groups.^9,12–14^. Throughout this paper, we will use the term ISUP grade. The ISUP grade suffers from several well-known limitations. For example, there is substantial disagreement in the grading using the Gleason scheme^15,14^. Furthermore, although the Gleason growth patterns have seen significant updates and additions since their inception in the 1960s, they remain relatively coarse descriptors of tissue morphology. As such, the prognostic potential of more fine-grained morphological features has been underexplored. We hypothesize that artificial intelligence, and more specifically deep learning, has the potential to discover such information and unlock the true prognostic value of morphological assessment of cancer. Specifically, we developed a deep learning system (DLS), trained on H&E-stained histopathological tissue sections, yielding a score for the likelihood of early biochemical recurrence.

Deep learning is a recent new class of machine learning algorithms that encompasses models called neural networks. These networks are optimized using training data; images with labels, such as recurrence information. From the training data, relevant features to predict the labels are automatically inferred. During development, the generalization of these features is tested on separated training data, which is not used for learning. Afterwards, a third independent set of data, the test set, is used to ensure generalization. Since features are inferred, handcrafted feature engineering is not needed anymore to develop machine learning models. Neural networks are the current state-of-the-art in image classification^16^.

Deep learning has previously been shown to find visual patterns to predict genetic mutations from morphology, for example, in lymphoma^17^ and lung cancer^18^. Additionally, deep learning has been used for feature discovery in colorectal cancer^19^ and intrahepatic cholangiocarcinoma^20^ using survival data. Although deep learning has been used with biochemical recurrence data on prostate cancer, Leo et al.^21^. assumed manual feature selection beforehand, strongly limiting the extent of new features to be discovered. Yamamoto et al.^22^. used whole slide images and a deep-learning-based encoding of the slides to tackle the slides’ high resolution. They leverage classical regression techniques and support-vector machine models on these encodings. The deep learning model was not directly trained on the outcome, limiting the feature discovery in this work as well.

A common critique of deep learning is its black-box nature of the inferred features^23^. Especially in the medical field, decisions based on these algorithms should be extensively validated and be explainable. Besides making the algorithms’ prediction trustworthy and transparent, from a research perspective, it would be beneficial to visualize the data patterns which the model learned, allowing insight into the inferred features. We can visualize the patterns learned by the network leveraging a new technique called Automatic Concept Explanations (ACE)^24^. ACE clusters patches of the input image using their intermediate inferred features showing common patterns inferred by the network. We were interested in finding these common concepts over a range of images to unravel patterns that the model has identified.

This study aimed to use deep learning to develop a new prognostic biomarker based on tissue morphology for recurrence in patients with prostate cancer treated by radical prostatectomy. As training data, we used a nested case-control study^25^. This study design ensured we could evaluate whether the network learned differentiating patterns independent of Gleason patterns. The prognostic biomarker provides a strong correlation with biochemical recurrence in two sets (*n* = 182 and *n* = 204) from separate institutions. Furthermore, the Automatic Concept-based Explanations provided tissue patterns interpretable by our pathologist.

## Methods

### Cohorts

Two independent cohorts of patients who underwent prostatectomy for clinically localized prostate cancer were used in this study. Patients were treated at either the Johns Hopkins Hospital in Baltimore or New York Langone Medical Centre. Both cohorts were accessed via the Prostate Cancer Biorepository Network^26^. The Johns Hopkins University School of Medicine Institutional Review Board and The New York University School of Medicine Institutional Review Board provided ethical regulatory approval for collection and disbursement of data and materials from the respective institutions. The need for acquiring informed consent was waived by the institutional ethical review boards.

For the development of the novel deep-learning-based biomarker (further referred to as DLS biomarker), we used a nested case-control study of patients from Johns Hopkins. This study consists of 524 matched pairs (724 unique patients) containing four tissue spots per patient. They were sampled from 4860 prostate cancer patients with clinically localized prostate cancer who received radical retropubic prostatectomy between 1993 and 2001. Men were routinely checked after prostatectomy at 3 months and at least yearly thereafter. Surveillance for recurrence was conducted using digital rectal examination and measurement of serum PSA concentration. Patients were followed for outcome until 2005, with a median follow-up of 4.0 years. The outcome was defined as recurrence, based on biochemical recurrence (serum PSA > 0.2 ng/mL on 2 or more occasions after a previously undetectable level after prostatectomy), or events indicating biochemical recurrence before this was measured; local recurrence, systemic metastases, or death from prostate cancer. Controls were paired to cases with recurrence using incidence density sampling^27^. For each case, a control was selected who had not experienced recurrence by the date of the case’s recurrence and was additionally matched based on age at surgery, race, pathologic stage, and Gleason sum in the prostatectomy specimen based on the pathology reports. Given the incidence density sampling of controls, some men were used as controls for multiple cases, and some controls developed recurrence later and became cases for that time period.

The TMA spots were cores (0.6 mm in diameter) from the highest-grade tumour nodule. Random subsamples were taken in quadruplicate for each case. The whole slides were scanned using a Hamamatsu NanoZoomer-XR slide scanner at 0.23 μm/px. TMA core images were extracted using QuPath (v0.2.3^28^,). We discarded analysis of cores with <25% tissue. The cores were manually checked (HP) for prostate cancer, excluding 535 cores without clear cancer cells present in the TMA cross-section, resulting in a total of 2343 TMA spots. The nested case-control set was split based on the matched pairs into a development set (268 unique pairs), and a test set (91 pairs); the latter was used for evaluation only. We leveraged cross-validation by subdividing the development into three folds to tune the models on different parts of the development set. We divided paired patient, randomly, keeping into account the distribution of the matched variables. The random assignment was done using the scikit-multilearn package^29^, specifically the ‘IterativeStratification’ method in ‘skmultilearn.model_selection’. After splitting the dataset into training and test, we split the training dataset into three folds using the same method for the cross-validation.

To validate the DLS biomarker on a fully independent external set, we used the cohort from New York Langone Medical Centre. This external validation cohort consists of 204 patients with localized prostate cancer treated with radical prostatectomy between 2001 and 2003. Patients were followed for outcome until 2019, with a median follow-up of 5 years. Biochemical recurrence was defined as either a single PSA measurement of ≥0.4 ng/m or PSA level of ≥0.2 ng/ml followed by increasing PSA values in subsequent follow-up. Cores were sampled from the largest tumour focus or any higher-grade focus (>3 mm). Subsamples were taken in quadruplicate for each case. Images were scanned using a Leica Aperio AT2 slide scanner at 0.25 μ/px.

### Model details

For developing the convolutional neural networks (CNNs) we used PyTorch^30^. As an architecture, we used ResNet50-D^31^ pretrained on ImageNet from PyTorch Image Models^32^. We used the Lookahead optimizer^33^ with RAdam^34^, with a learning rate of 2e-4 and mini-batch size of 16 images. We used weight decay (7e-3), and a drop-out layer (*p* = 0.15) before the final fully-connected layer. We used EfficientNet-style^35^ dropping of residual connections (*p* = 0.3) as implemented in PyTorch Image Models. We used Bayesian Optimization to find the optimal values (See Supplementary Notes 1 for details about the searchspace).

We resized the TMAs to 1.0 mu/pixel spacing and cropped to 768 × 768 pixels. Extensive data augmentations were used to promote generalization. The transformations were: flipping, rotations, warping, random crop, HSV colour augmentations, jpeg compression, elastic transformations, Gaussian blurring, contrast alterations, gamma alterations, brightness alterations, embossing, sharpening, Gaussian noise and cutout^36^. Augmentations were implemented using albumentations^37^ and fast.ai^38^.

TMA spots from cases experiencing recurrence were assigned a value of 0–4, depending on the year on which the first event, either biochemical recurrence, metastases, or prostate cancer-related death, was recorded, with 0 meaning recurrence within a year, four meaning after 4+ years. TMA spots from cases without an event were also assigned the label 4.

We validated the model on the development validation fold each epoch with a moving average of the weights from five subsequent epochs. We used the concordance index as a metric to decide which model performed the best.

**Fig. 1:**
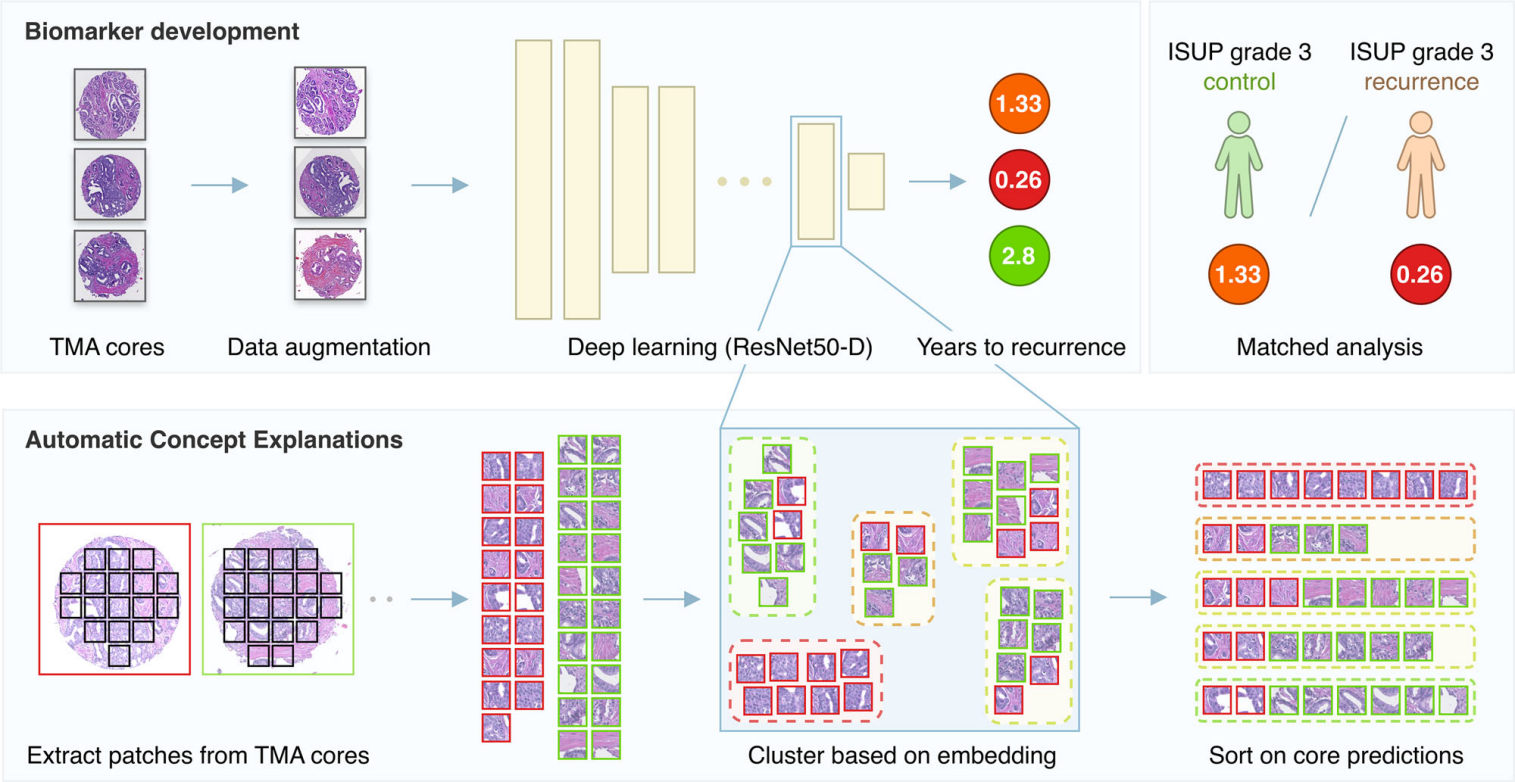
Overview of the methods summarizing the biomarker development and the Automatic Concept Explanations (ACE) process. Cores were extracted from TMA slides and used to train a neural network to predict the years to biochemical recurrence. On the nested case-control test set, a matched analysis was performed. For ACE, patches were generated from the cores, inferenced through the network and clustered based on their intermediate features.

As the final prediction at the patient level, the TMA spot with the highest score was used. The final DLS consists of an ensemble of 15 convolutional neural networks. Using cross-validation as described above, 15 networks were trained for each fold, of which the five best performing were used for the DLS. See Fig. 1 for a graphical overview of the methods, further details can be found in the Supplementary Methods.

### Statistics and reproducibility

For primary analysis of the nested case-control study, odds ratios (OR) and 95% confidence intervals (CI) were calculated using conditional logistic regression, following Dluzniewski et al.^39^. Due to the study design, calculating hazard ratios using a Cox proportional hazard regression is not appropriate. For the primary analysis, the continuous DLS marker was given as the only variable. For a secondary analysis, we added the non-matched variables PSA, positive surgical margins, and a binned indicator variable for year of surgery. Since matching was done on Gleason sum, and our goal was to identify patterns beyond currently used Gleason patterns, we corrected for the residual differences of the ISUP grade between cases and control (see Table 1). A correction was performed by adding a continuous covariate since, due to the small differences, an indicator covariate did not converge. Analysis was done using the lifelines Python package (v. 0.25.10)^40^ with Python (v. 3.7.8). *P*-values were calculated as a Wald test per single parameter. Since the DLS predicts the time-to-recurrence, high values indicate a low probability of recurrence. We multiplied the DLS output by −1 to make the analysis more interpretable. For three patients (1 from the Johns Hopkins cohort and 2 from the New York Langone cohort), PSA values were missing and were therefore replaced by the median.

**Table 1:** Baseline characteristics of test set and development set from the John Hopkins Hospital, prostate cancer recurrence cases and controls, men who underwent radical prostatectomy for clinically localized disease between 1993 and 2001.

	Development set			Test set		
	Recurrence cases	No events cases	*P*	Recurrence cases	Controls*	*P*
***N***	368	135		91	91	
**Age, mean (SD)**	58.9 (6.2)	59.3 (6.3)	*p* = 0.540	58.4 (6.1)	58.3 (6.3)	Matched
**preop. PSA (ng/mL), mean (SD)**	12.3 (10.0)	10.1 (7.5)	*p* = 0.010	12.3 (10.8)	10.5 (7.7)	*p* = 0.195
**Race,** ***n*** **(%)**			*p* = 0.599			Matched
White	327 (88.9)	120 (88.9)		72 (79.1)	75 (82.4)	
Black or African–American	32 (8.7)	14 (10.4)		12 (13.2)	10 (11.0)	
Other	9 (2.4)	1 (0.7)		7 (7.7)	6 (6.6)	
**Pathological stage**			*p* = 0.107			Matched
pT2	43 (11.7)	25 (18.5)		20 (22.0)	19 (20.9)	
pT3a	199 (54.1)	63 (46.7)		50 (54.9)	51 (56.0)	
pT3b or N1	126 (34.2)	47 (34.8)		21 (23.1)	21 (23.1)	
**Gleason sum prostatectomy (%)**			*p* = 0.179			Matched
6	38 (10.3)	25 (18.5)		20 (22.0)	23 (25.3)	
7	233 (63.3)	76 (56.3)		51 (56.0)	50 (54.9)	
8+	97 (26.4)	34 (25.2)		20 (22.0)	18 (19.8)	
**ISUP grade,** ***n*** **(%)**			*p* = 0.002			*p* = 0.851
1	38 (10.3)	25 (18.5)		20 (22.0)	23 (25.3)	
2	140 (38.0)	61 (45.2)		35 (38.5)	38 (41.8)	
3	93 (25.3)	15 (11.1)		16 (17.6)	12 (13.2)	
4	49 (13.3)	21 (15.6)		13 (14.3)	10 (11.0)	
5	48 (13.0)	13 (9.6)		7 (7.7)	8 (8.8)	
**Positive surgical margins**	140 (38.1)	24 (17.8)	*p* < 0.001	36 (39.6)	20 (22.0)	*p* = 0.016
**Mean year of surgery**	1997.0 (2.3)	1995.5 (2.3)	*p* < 0.001	1997 (2.3)	1995 (2.1)	*p* < 0.001

*Due to the nested case-control nature, some controls could have a biochemical recurrence, but always later than their matched case.

For primary analysis of the New York Langone cohort, we calculated hazard ratios (HR) using a Cox proportional hazards regression. We report a secondary multivariable analysis including indicator variables for relevant clinical covariates, Gleason sum, pathological stage, and surgical margin status. We tested the proportional hazards assumption as satisfactory (every *p*-value > 0.01) using the Pearson correlation between the residuals and the rank of follow-up time. Kaplan–Meier plots were generated for the New York Langone cohort. Due to the nested case-control design for the Johns Hopkins set, this set could not be visualized in a Kaplan–Meier plot.

### Automatic concept explanations

To generate concepts, we picked the best performing single CNN from the DLS based on its validation set fold. We used a combination of the methods of Yeh et al., 2020^41^ and Ghorbani et al., 2019^24^.

We tiled the TMA images into 256 × 256 patches within the tissue, discarding patches with >50% whitespace. These patches were padded to the original input shape of the CNN (768 × 768 pixels). The latent space of layer 42 of 50 was saved for each tile. Afterwards, we used PCA (50 components) to lower the dimensionality and then performed k-means (*k* = 15) to cluster the latent spaces.

In contrast to Yeh et al. and Ghorbani et al., we did not sort the concepts on completeness of the explanations or importance for prediction of individual samples. We sorted the concepts to find interesting new patterns related to recurrence across images by ranking the concepts based on the DLS score of the TMA spot from which they originated.

For each concept, 25 examples were randomly picked and visually inspected by a pathologist (JvI), with a special interest in uropathology, blinded to the case characteristics and prediction of the network.

### Reporting summary

Further information on research design is available in the Nature Research Reporting Summary linked to this article.

## Results

The DLS system was developed on the Johns Hopkins cohort with 2343 TMA spots of 685 included unique patients (39 patients were excluded due to insufficient tumour amount in the cores). Four hundred ninety-two patients were recurrence cases (72%). The 685 included patients were split into a development set of 503 unique patients and a test set of 91 matched pairs of cases and controls (182 unique patients).

In the external validation cohort, 38 out of the 204 patients (19%) had biochemical recurrence after complete remission, PSA nadir after 3 months post-prostatectomy. From the 204 patients, 620 TMA spots were included. Clinical characteristics of the cohorts can be found in Table 1 and Table 2.

**Table 2:** Baseline characteristics of the cohort from New York Langone hospital, prostate cancer recurrence cases and controls, men who underwent radical prostatectomy between 2001 and 2003.

	Recurrence cases	Controls	*P*
*N*	38	166	
**preop. PSA (ng/mL), mean (SD)**	11.6 (11.5)	6.7 (3.9)	*p* = 0.014
**Age, mean (SD)**	61.7 (8.9)	60.3 (6.6)	*p* = 0.359
**Race,** ***n*** **(%)**			*p* = 0.401
African–American	2 (5.3)	4 (2.4)	
Asian	2 (5.3)	3 (1.8)	
Caucasian	33 (86.8)	144 (86.7)	
Not reported	0 (0)	2 (1.2)	
Other	1 (2.6)	13 (7.8)	
**Pathological stage,** ***n*** **(%)**			*p* < 0.001
pT2a	0 (0)	12 (7.2)	
pT2b	3 (7.9)	5 (3.0)	
pT2c	16 (42.1)	114 (68.7)	
pT3a	10 (26.3)	27 (16.3)	
pT3b	9 (23.7)	8 (4.8)	
**ISUP grade,** ***n*** **(%)**			*p* < 0.001
1	3 (7.9)	67 (40.4)	
2	13 (34.2)	76 (45.8)	
3	6 (15.8)	13 (7.8)	
4	5 (13.2)	3 (1.8)	
5	11 (28.9)	7 (4.2)	
**Surgical Margins,** ***n*** **(%)**			*p* = 0.060
Focal	10 (26.3)	20 (12.0)	
Free of tumour	27 (71.1)	144 (86.7)	
Widespread	1 (2.6)	2 (1.2)	

The DLS marker showed a strong association in the primary analyses on the test set of the Johns Hopkins cohort with an OR of recurrence of 3.28 (95% CI 1.73–6.23; *p* < 0.005) per unit increase, with DLS system continuous output ranging from 0–3, with two cases below 0 (−0.27 and −0.24) (Table 3).

**Table 3:** Conditional logistic regression analyses of the Johns Hopkins test set.

Covariate	Matched analysis Johns Hopkins (OR)^a^	Multivariate analysis Johns Hopkins (OR)
**Biomarker**	3.28 (CI 1.73–6.23; *p* < 0.005)	3.32 (CI 1.63–6.77; *p* = 0.001)
**preop. PSA (ng/mL)**		1.04 (CI 0.99–1.10; *p* = 0.10)
**Surgical margins (pos)**		1.69 (CI 0.69–4.18; *p* = 0.25)
**ISUP grade (cont.)**^**b**^		1.34 (CI 0.64–2.82; *p* = 0.44)
**Mean year of surgery**		
1992–1994 (*n* = 75)		1.0
1994–1997 (*n* = 55)		3.35 (CI 1.13–9.91; *p* = 0.03)
1997–2001 (*n* = 52)		8.22 (CI 2.38–28.37; *p* = 0.0009)

^a^Cases and controls were matched on age at surgery, race, pathologic stage, and Gleason sum in the prostatectomy specimen.

^b^ The ISUP grade covariate was added to correct for the residual differences left after matching cases with controls on prostatectomy Gleason sum.

In addition, for the John Hopkins cohort, we checked for confounding by ISUP grade, PSA level at diagnosis, positive surgical margins, and year of prostatectomy. Neither covariate was found to bias the estimates of effect substantially. The biomarker maintained a strong correlation of OR 3.32 (CI 1.63–6.77; *p* = 0.001) per unit increase, adjusting for these factors and the continuous term for the residual difference between cases and controls in the ISUP grade.

**Fig. 2:**
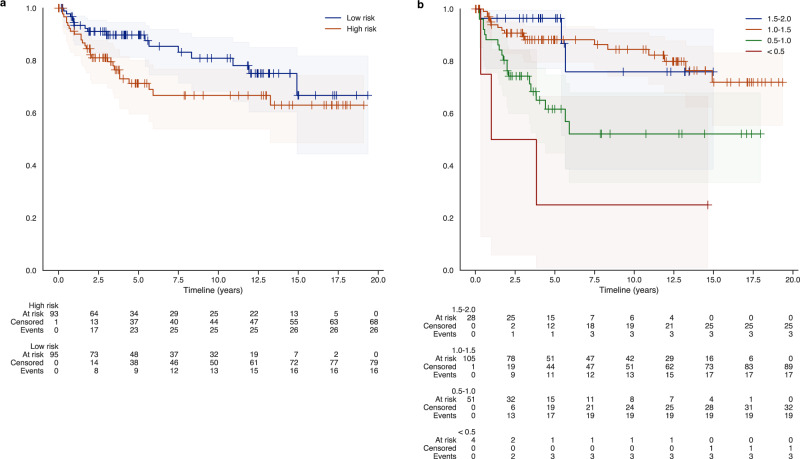
Kaplan–Meier plot for New York Langone external validation cohort. Groups were separated using the median DLS biomarker score in this cohort (**a**) and using four thresholds (**b**).

In the univariable analysis, the DLS marker was strongly associated with recurrence in the New York Langone external validation cohort with an HR of 5.78 (95% CI 2.44–13.72; *p* < 0.005) per unit increase. In the multivariate model, including ISUP grade and the other prognostic indicators in addition to the DLS biomarker, the DLS biomarker was still strongly associated with recurrence with an HR of 3.02 (CI 1.10–8.29; *p* = 0.03) per unit increase (Table 4). Kaplan–Meier curves based on a median cut-off, and four-group categorization, show a clear separation of the low-risk and high-risk groups (Fig. 2).

**Table 4:** Cox proportional hazard analyses of New York Langone external validation cohort.

Covariate	Univariate analysis NYU (HR)	Multivariate analysis NYU (HR)
**Biomarker**	4.79 (CI 2.09–10.96; *p* = 0.0002)	3.02 (CI 1.10–8.29; *p* = 0.03)
**preop. PSA (ng/mL)**		1.07 (CI 1.02–1.12; *p* = 0.004)
**ISUP grade**		
1		1.0
2		2.64 (CI 0.73–9.58; *p* = 0.14)
3		8.74 (CI 2.16–35.30; *p* = 0.00)
4		12.78 (CI 2.82–57.91; *p* = 0.00)
5		9.60 (CI 2.32–39.69; *p* = 0.00)
**Pathological stage**		
pT2a + b		1.0
pT2c		1.02 (CI 0.27–3.80; *p* = 0.98)
pT3a		1.26 (CI 0.28–5.67; *p* = 0.77)
pT3b		2.77 (CI 0.66–11.62; *p* = 0.16)
**Surgical margins**		
Free		1.0
Focal		2.13 (CI 0.76–5.96; *p* = 0.15)
Widespread		0.20 (CI 0.01–3.39; *p* = 0.27)

**Fig. 3:**
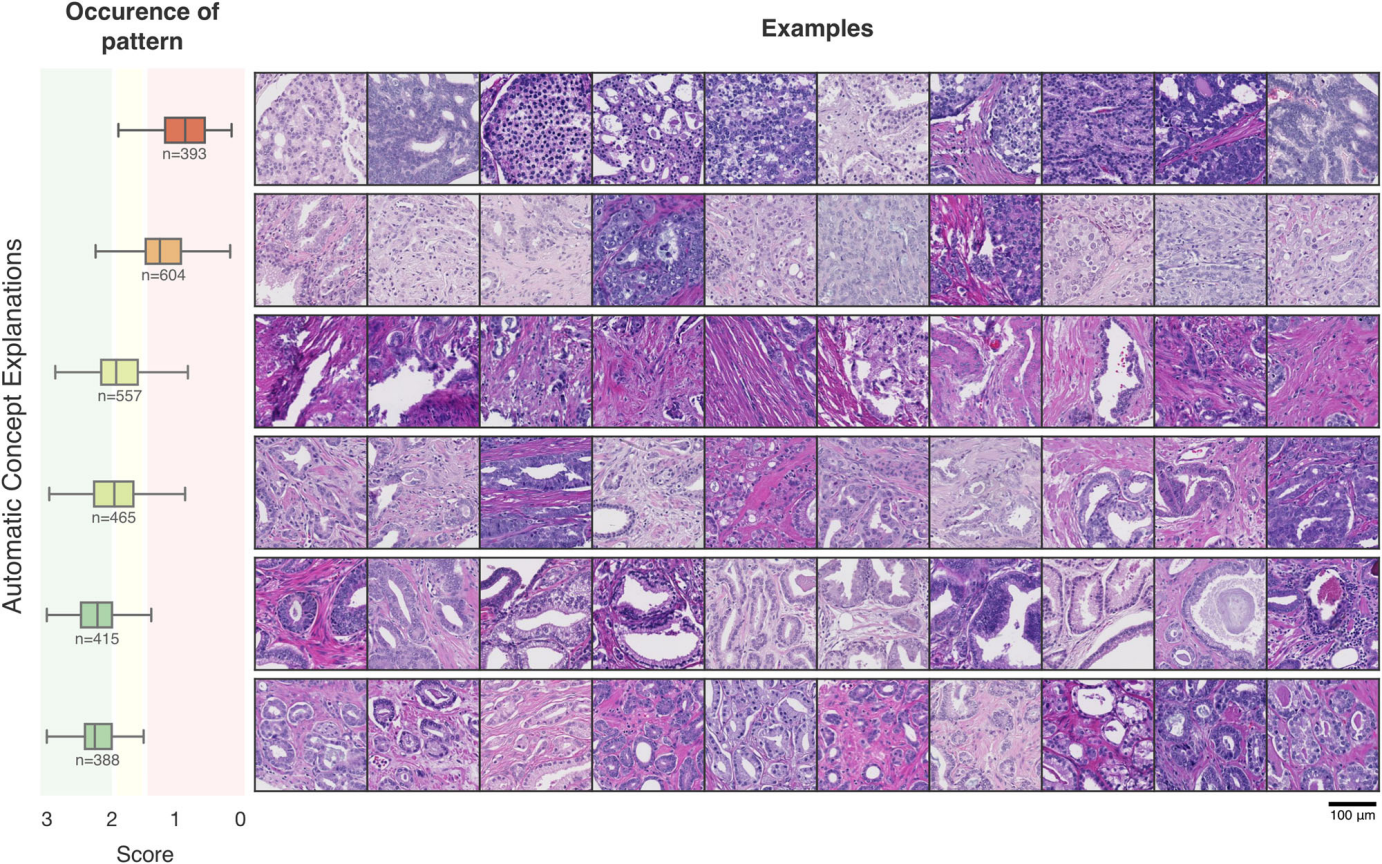
Examples of automatic concepts explanations. Concepts were sorted by their average score for the cores in which the pattern occurs. Showing the two most benign concepts, two intermediate and two aggressive concepts. The boxes show the quartiles of the concept predictions while the whiskers extend to show the rest of the distribution except for outlier points that lie below the 25% or above 75% of the data, by 1.5 times the interquartile range. Green, yellow and red shaded areas indicate 33%, 66% percentiles. See the Supplementary Notes 2 for all concepts.

Automatic Concept Explanations provided semantically meaningful concepts (Fig. 3). Concepts were identified that correlated with either a relatively rapid or slow biochemical recurrence. Visual inspection by JvI reveals that generally, the concepts with adverse behaviour show mainly Gleason pattern 4 and some Gleason pattern 5, with cribriform configuration in TMAs within the concepts with most adverse behaviour. The two intermediate concepts show mainly stroma and less aggressive growth patterns. The two concepts predicted to be part of late recurrence cases show mainly Gleason 3 patterns, with readily recognizable well-formed glands. See the Supplementary Notes 2 for a detailed analysis.

## Discussion

We have developed a deep-learning-based morphological biomarker for the prediction of prostate cancer biochemical recurrence based on prostatectomy tissue microarrays. Using a nested case-control study, we trained convolutional neural networks end-to-end with biochemical recurrence data. The DLS marker provides a continuous score based on the speed of biochemical recurrence it perceived. The DLS marker had an OR of 3.32 (CI 1.63–6.77; *p* = 0.001) per unit increase for the test set, and an HR of 3.02 (CI 1.10–8.29; *p* = 0.03) per unit increase for the external validation set. These findings support our hypothesis that there is more morphological information in the tissue besides the ISUP grade.

In the Kaplan–Meier plot (Fig. 2), the biomarker especially seems able to separate men with relatively rapid recurrence from men without (<5 years). However, we hypothesize that the decreased long-term separation in those survival curves is less due to the training cohort containing a median follow-up for 4 years. Furthermore, we choose to group patients together with >4 years of no biochemical recurrence, this limits the model’s capabilities to differentiate patients with very late recurrence. Additionally, due to the limitations of the morphology of the present tumour to inform about long-term outcomes (e.g., cells that escaped the primary tumour may subsequently acquire genomic changes that influence recurrence). Furthermore, it should be noted that the number of at-risk patients was small at these long-term time points.

The nested case-control study contained follow-up information in timespans of years, this limited the use of survival based loss functions^42^. When more granular follow-up information is at hand, future work could investigate usage of Cox regression based loss functions to better leverage the information of the clinical cohort.

The DLS marker showed strong and similar association in both cohorts prepared at different pathology laboratories, which supports the robustness to differences in tissue preparation, staining protocols and scanners.

We showed that Automatic Concept Explanation may be helpful to find concepts correlated with good and poor prognosis. The most discriminatory concepts followed the morphological patterns of Gleason grading. Well-defined prostate cancer glands were predicted to undergo biochemical recurrence later than disorganized sheets of prostate cancer cells. These concepts support the DLS system capturing the expected morphological patterns in support of the validity of the DLS approach.

This study focused on the use of deep learning to automatically discover features relevant for biochemical recurrence prediction. Compared to before-mentioned studies on prostate cancer prognostics models^21,22^, as far as we know, we report the first paper to directly optimize a neural network from prostatectomy tissue towards biochemical recurrence. Additionally, we report that training towards the biochemical recurrence endpoint results in patterns in the networks’ features aligning with the ISUP grading.

In the increasing digitalisation of pathology labs, our DLS marker may be applied on digitally chosen regions of interest. Our marker is trained on tissue microarray spots that were selected at the highest-grade cancer focus. Furthermore, it has to be noted that a TMA core allows for only limited assessment of the overall prostate cancer growth patterns. Since these tissue cores represent only limited samples from what is usually a much larger tumour lesion, the potential more aggressive patterns may still be present outside of the chosen regions, including regions of potential extraprostatic extension and perineural invasion. Validation will need to be done on entire prostatectomy sections and across cancer foci.

There have been improvements to prostate cancer grading^11,13^, and recently the cribriform pattern is suggested to be important for prognostics^14,43^. However, the evaluation of this pattern can show a range of inter-observer variability^44^, although a recent consensus approach could help decrease this variability^45^. Although we certainly have to keep in mind all the before-mentioned limitations, our findings are in line with outcomes concerning adverse behaviour in earlier work. The DLS system identified a concept that consisted of fields with cribriform-like growth patterns. This cribriform-like growth pattern was found to be part of the concept that was most associated with early recurrent cases.

The results in this study are limited to newer insights of prostate cancer growth, information on cribriform-growth and intraductal carcinoma were not readily available for use in the multivariate analysis, although the external validation cohort was graded using the 2005 ISUP consensus^46^ partly encoding the presence of cribriform growth inside the ISUP grade.

Although biochemical recurrence is a common endpoint to study prostate cancer progression, a clinical utility would be mostly found in assessing time-to-metastases or death. However, time-wise, they are typically significantly further separated from the surgical event, making it harder to identify relationships between tissue morphology and these endpoints. Nevertheless, we would like to investigate them in the future.

## Conclusions

In summary, we have developed a deep-learning-based visual biomarker for prostate cancer recurrence based on tissue microarray hotspots of prostatectomies. The DLS marker provides a continuous score predicting the speed of biochemical recurrence. We obtained an odds ratio of 3.32 (CI 1.63–6.77; *p* = 0.001) for a nested case-control study from Johns Hopkins Hospital, matched on Gleason sum on other factors. Additionally, we obtained an HR of 3.02 (CI 1.10–8.29; *p* = 0.03) for an external validation cohort from the New York Langone hospital, adjusted for ISUP grade, pathological stage, preoperative PSA concentration, and surgical margins status. Thus, this visual biomarker may provide prognostic information in addition to the current morphological ISUP grade.

## Supplementary information

Description of Additional Supplementary Files

Supplementary Data 1

Supplementary Data 2

Supplementary Data 3

Supplementary Data 4

Supplementary Information

Reporting Summary

## Data Availability

The data that support the findings of this study are available from the Prostate Cancer Biorepository Network^26^ but restrictions apply to the availability of these data, which were used under license for the current study, and so are not publicly available. Data are however available from the authors upon reasonable request and with permission of the Prostate Cancer Biorepository Network^26^. Source data for Figs. 2 a, b, and 3 and Supplementary Fig. 1 can be accessed as Supplementary Data 1, 2, 3 and 4, respectively.

## Code availability

The code to replicate the DLS biomarker can be found at https://zenodo.org/record/6480481^47^.

## References

[1] Sung, H. et al. Global cancer statistics 2020: GLOBOCAN estimates of incidence and mortality worldwide for 36 cancers in 185 countries. *CA Cancer J. Clin.*10.3322/caac.21660 (2021). 10.3322/caac.21660 33538338

[2] Grossman DC (2018). Screening for Prostate Cancer: US preventive services task force recommendation statement. JAMA.

[3] Heijnsdijk EAM (2018). Summary statement on screening for prostate cancer in Europe. Int J Cancer.

[4] Goonewardene SS, Phull JS, Bahl A, Persad RA (2014). Interpretation of PSA levels after radical therapy for prostate cancer. Trends Urol. Men S Health.

[5] Amling CL (2000). Long-term hazard of progression after radical prostatectomy for clinically localized prostate cancer: continued risk of biochemical failure after 5 years. J Urol..

[6] Freedland SJ (2005). Risk of prostate cancer–specific mortality following biochemical recurrence after radical prostatectomy. JAMA.

[7] Han M, Partin AW, Pound CR, Epstein JI, Walsh PC (2001). Long-term biochemical disease-free and cancer-specific survival following anatomic radical retropubic prostatectomy. The 15-year Johns Hopkins experience. Ur. Clin. North Am..

[8] Van den Broeck T (2019). Prognostic value of biochemical recurrence following treatment with curative intent for prostate cancer: a systematic review. Eur. Urol..

[9] Epstein JI (2016). The 2014 International Society of Urological Pathology (ISUP) consensus conference on Gleason grading of prostatic carcinoma. Am. J. Surg. Pathol..

[10] Mottet N (2021). EAU-EANM-ESTRO-ESUR-SIOG Guidelines on Prostate Cancer—2020 Update. Part 1: screening, diagnosis, and local treatment with curative intent. Eur. Urol..

[11] Epstein JI (2010). An update of the Gleason grading system. J. Urol..

[12] Pierorazio PM, Walsh PC, Partin AW, Epstein JI (2013). Prognostic Gleason grade grouping: data based on the modified Gleason scoring system. BJU Int.

[13] Epstein JI (2016). A Contemporary Prostate Cancer Grading System: a validated alternative to the Gleason score. Eur. Urol.

[14] van Leenders GJLH (2020). The 2019 International Society of Urological Pathology (ISUP) consensus conference on Grading of prostatic carcinoma. Am. J. Surg. Pathol..

[15] Ozkan TA (2016). Interobserver variability in Gleason histological grading of prostate cancer. Scand. J. Urol..

[16] Krizhevsky A, Sutskever I, Hinton GE (2012). Imagenet classification with deep convolutional neural networks. Adv. Neural Inf. Process. Syst..

[17] Swiderska-Chadaj, Z., Hebeda, K. M., van den Brand, M. & Litjens, G. Artificial intelligence to detect MYC translocation in slides of diffuse large B-cell lymphoma. *Virchows Arch*. 10.1007/s00428-020-02931-4 (2020). 10.1007/s00428-020-02931-4 PMC8448690 32979109

[18] Coudray N (2018). Classification and mutation prediction from non–small cell lung cancer histopathology images using deep learning. Nat. Med..

[19] Wulczyn E (2021). Interpretable survival prediction for colorectal cancer using deep learning. NPJ Digit. Med.

[20] Muhammad, H. et al. EPIC-Survival: End-to-end part inferred clustering for survival analysis, featuring prognostic stratification boosting. *arXiv*10.48550/arXiv.2101.11085 (2021).

[21] Leo, P. et al. Computer extracted gland features from H&E predicts prostate cancer recurrence comparably to a genomic companion diagnostic test: a large multi-site study. *Npj Precis. Oncol.*10.1038/s41698-021-00174-3 (2021). 10.1038/s41698-021-00174-3 PMC8093226 33941830

[22] Yamamoto Y (2019). Automated acquisition of explainable knowledge from unannotated histopathology images. Nat. Commun..

[23] Rudin C (2019). Stop explaining black box machine learning models for high stakes decisions and use interpretable models instead. Nat. Mach. Intell..

[24] Ghorbani, A., Wexler, J., Zou, J. & Kim, B. Towards automatic concept-based explanations. *arXiv*10.48550/arXiv.1902.03129 (2019).

[25] Toubaji A (2011). Increased gene copy number of ERG on chromosome 21 but not TMPRSS2-ERG fusion predicts outcome in prostatic adenocarcinomas. Mod. Pathol..

[26] PCBN. *Prostate Cancer Biorepository Network* https://prostatebiorepository.org/ (2021).

[27] Wang M-H, Shugart YY, Cole SR, Platz EA (2009). A simulation study of control sampling methods for nested case-control studies of genetic and molecular biomarkers and prostate cancer progression. Cancer Epidemiol. Biomarkers Prev..

[28] Bankhead P (2017). QuPath: Open source software for digital pathology image analysis. Sci. Rep..

[29] Szymanski P, Kajdanowicz T (2019). Scikit-multilearn: a scikit-based Python environment for performing multi-label classification. J. Mach. Learn. Res..

[30] Paszke, A. et al. PyTorch: An imperative style, high-performance deep learning library. *arXiv*10.48550/arXiv.1912.01703 (2019).

[31] He, T. et al. Bag of tricks for image classification with convolutional neural networks. In *Proc. IEEE/CVF Conference on Computer Vision and Pattern Recognition*. 558–567 (IEEE, 2019).

[32] Wightman, R. PyTorch image models. *GitHub*10.5281/ZENODO.4414861 (2021).

[33] Zhang, M. R., Lucas, J., Hinton, G. & Ba J. Lookahead optimizer: k steps forward, 1 step back. *arXiv*10.48550/arXiv.1907.08610 (2019).

[34] Liu L., et al. On the variance of the adaptive learning rate and beyond. *arXiv*10.48550/arXiv.1908.03265 (2019).

[35] Tan, M. & Le, Q. Efficientnet: Rethinking model scaling for convolutional neural networks. *arXiv*10.48550/arXiv.1905.11946 (2019).

[36] DeVries, T. & Taylor, G. W. Improved regularization of convolutional neural networks with cutout. *arXiv*10.48550/arXiv.1708.04552 (2017).

[37] Buslaev A (2020). Albumentations: fast and flexible image augmentations. Information.

[38] Howard J, Gugger S (2020). Fastai: A layered API for deep learning. Information.

[39] Dluzniewski PJ (2012). Variation in IL10 and other genes involved in the immune response and in oxidation and prostate cancer recurrence. Cancer Epidemiol. Biomarkers Prev..

[40] Davidson-Pilon, C. et al. *CamDavidsonPilon/lifelines: 0.25.10*. Zenodo 10.5281/ZENODO.4579431 (2021).

[41] Yeh, C.-K. et al. On completeness-aware concept-based explanations in deep neural networks. *Adv. Neural Inf. Process. Syst.*10.48550/arXiv.1910.07969 (2020).

[42] Kvamme H, Borgan Ø, Scheel I (2019). Time-to-event prediction with neural networks and cox regression. J. Mach. Learn. Res..

[43] Hollemans E (2021). Cribriform architecture in radical prostatectomies predicts oncological outcome in Gleason score 8 prostate cancer patients. Mod. Pathol..

[44] van der Slot MA (2021). Inter-observer variability of cribriform architecture and percent Gleason pattern 4 in prostate cancer: relation to clinical outcome. Virchows Arch..

[45] van der Kwast, T. H. et al. ISUP consensus definition of cribriform pattern prostate cancer. *Am. J. Surg. Pathol*. 10.1097/PAS.0000000000001728 (2021). 10.1097/PAS.0000000000001728 33999555

[46] Epstein JI, Allsbrook WC, Amin MB, Egevad LL, ISUP Grading Committee. (2005). The 2005 International Society of Urological Pathology (ISUP) consensus conference on Gleason grading of prostatic carcinoma. Am. J. Surg. Pathol..

[47] Pinckaers, H. *Source Code for “Predicting Biochemical Recurrence of Prostate Cancer with Artificial Intelligence”*. 10.5281/zenodo.6480481 (2022). 10.1038/s43856-022-00126-3 PMC9177591 35693032

