# Supplementary material for: Predicting biochemical recurrence of prostate cancer with artificial intelligence
Source: Commun Med (Lond). 2022 Jun 8;2:64. doi: 10.1038/s43856-022-00126-3 (PMC9177591; doi:10.1038/s43856-022-00126-3)
Supplement: Supplementary file 1 — Description of Additional Supplementary Files [file 43856_2022_126_MOESM1_ESM.pdf]

Description of Additional Supplementary Files

Supplementary Data 1: Source data for Figures 2a. The column 'Event' denotes a biochemical failure event (1=event), 'Times' denotes the time in years to the event (when event=1) or to drop out of the study (when event=0). The column 'Risk group' corresponds to the high and low risk groups from the figure (high=1, low=0).

Supplementary Data 2: Source data for Figures 2b. The column 'Event' denotes a biochemical failure event (1=event), 'Times' denotes the time in years to the event (when event=1) or to drop out of the study (when event=0). The column 'Risk group' corresponds to the risk groups from the figure (<0.5=1, 0.5-1.0=2, 1.0-1.5=3, 1.5-2.0=4).

Supplementary Data 3: Source data for Figures 3. Every column contains information about a patch. The column 'cluster' contains which cluster the patch was assigned to, sorted on the average prediction of the TMA cores contained in the cluster, ascending order. The column 'score' denotes the prediction of the DLS model on the originating TMA core.

Supplementary Data 4: Source data for Supplementary Figure 1. Every column contains information about a patch. The column 'cluster' contains which cluster the patch was assigned to, sorted on the average prediction of the TMA cores contained in the cluster, ascending order. The column 'score' denotes the prediction of the DLS model on the originating TMA core.
