# Supplementary material for: Predicting biochemical recurrence of prostate cancer with artificial intelligence
Source: Commun Med (Lond). 2022 Jun 8;2:64. doi: 10.1038/s43856-022-00126-3 (PMC9177591; doi:10.1038/s43856-022-00126-3)
Supplement: Supplementary file 6 — Supplementary Information [file 43856_2022_126_MOESM6_ESM.docx]

**Supplementary Information**

**Predicting biochemical recurrence of prostate cancer after radical prostatectomy with artificial intelligence**

Hans Pinckaers, Jolique van Ipenburg, Jonathan Melamed*,* Angelo De Marzo, Elizabeth A. Platz, Bram van Ginneken, Jeroen van der Laak, Geert Litjens

**Supplementary Methods**

*Details about model development*

As an architecture, we used ResNet50-D[[1]](https://paperpile.com/c/4oaJCk/0C4uw) pretrained on ImageNet from PyTorch Image Models[[2]](https://paperpile.com/c/4oaJCk/R8rzj). We resized the TMAs to 1.0 mu/pixel spacing and cropped to 768x768 pixels. Extensive data augmentations were used to promote generalization. The transformations were: flipping, rotations, warping, random crop, HSV color augmentations, jpeg compression, elastic transformations, Gaussian blurring, contrast alterations, gamma alterations, brightness alterations, embossing, sharpening, Gaussian noise and cutout[[3]](https://paperpile.com/c/4oaJCk/pupvV). Augmentations were implemented by albumentations[[4]](https://paperpile.com/c/4oaJCk/Wwhua) and fast.ai[[5]](https://paperpile.com/c/4oaJCk/eqdzO).For developing the convolutional neural networks (CNNs) we used PyTorch[[6]](https://paperpile.com/c/4oaJCk/ZASvN). As an architecture, we used ResNet50-D[[1]](https://paperpile.com/c/4oaJCk/0C4uw) pretrained on ImageNet from PyTorch Image Models[[2]](https://paperpile.com/c/4oaJCk/R8rzj). We used the Lookahead optimizer[[7]](https://paperpile.com/c/4oaJCk/9wkWS) with RAdam[[8]](https://paperpile.com/c/4oaJCk/uGUEw), with a learning rate of 2e-4 and mini-batch size of 16 images. We optimized the SmoothL1 loss function of PyTorch[4]. We used weight decay (7e-3), and a drop-out layer (p=0.15) before the final fully-connected layer. We used EfficientNet-style[[9]](https://paperpile.com/c/4oaJCk/ZG7CK) dropping of residual connections (p=0.3) as implemented in PyTorch Image Models. We used Bayesian Optimization to find the optimal values, see Supplemental Table 1 for all the hyperparameter ranges used.

We validated the model on the development validation fold each epoch with a moving average of the weights from 5 subsequent epochs. We used the concordance index as a metric to decide which model performed the best.

The individual output ranges of the neural networks in the ensemble of the DLS system lies between -1 and 5. The final DLS score is an average over all the network predictions on a single TMA core, the final output range then becomes 0-3.

*Details about Automatic Concept Explanations*

We tiled the TMA images into 256x256 patches within the tissue, discarding patches with more than 50% whitespace. These patches were padded to the original input shape of the CNN (768x768 pixels). The latent space of layer 42 of 50 was saved for each tile. Afterwards, we used PCA (50 components) to lower the dimensionality and then performed k-means (k=15) to cluster the latent spaces.

In contrast to Yeh *et al.* and Ghorbani *et al.*, we did not sort the concepts on completeness of the explanations or importance for prediction of individual samples. We sorted the concepts to find interesting new patterns related to recurrence across images by ranking the concepts based on the DLS score of the TMA spot from which they originated.

**Supplementary Notes 1**

| **Supplementary Table 1:** Hyperparameter searchspace using Bayesian Optimization. | | |
| --- | --- | --- |
| **Hyperparameter** | **Minimum** | **Maximum** |
| **Learning rate** | 3e-5 | 1e-2 |
| **Weight decay** | 1e-5 | 2e-4 |
| **Batch size** | 4 | 16 |
| **Drop rate of residual connections** | 0 | 0.8 |
| **Dropout rate** | 0 | 0.8 |
| **Number of recurrence year groupings** | 3 | 6 |

**
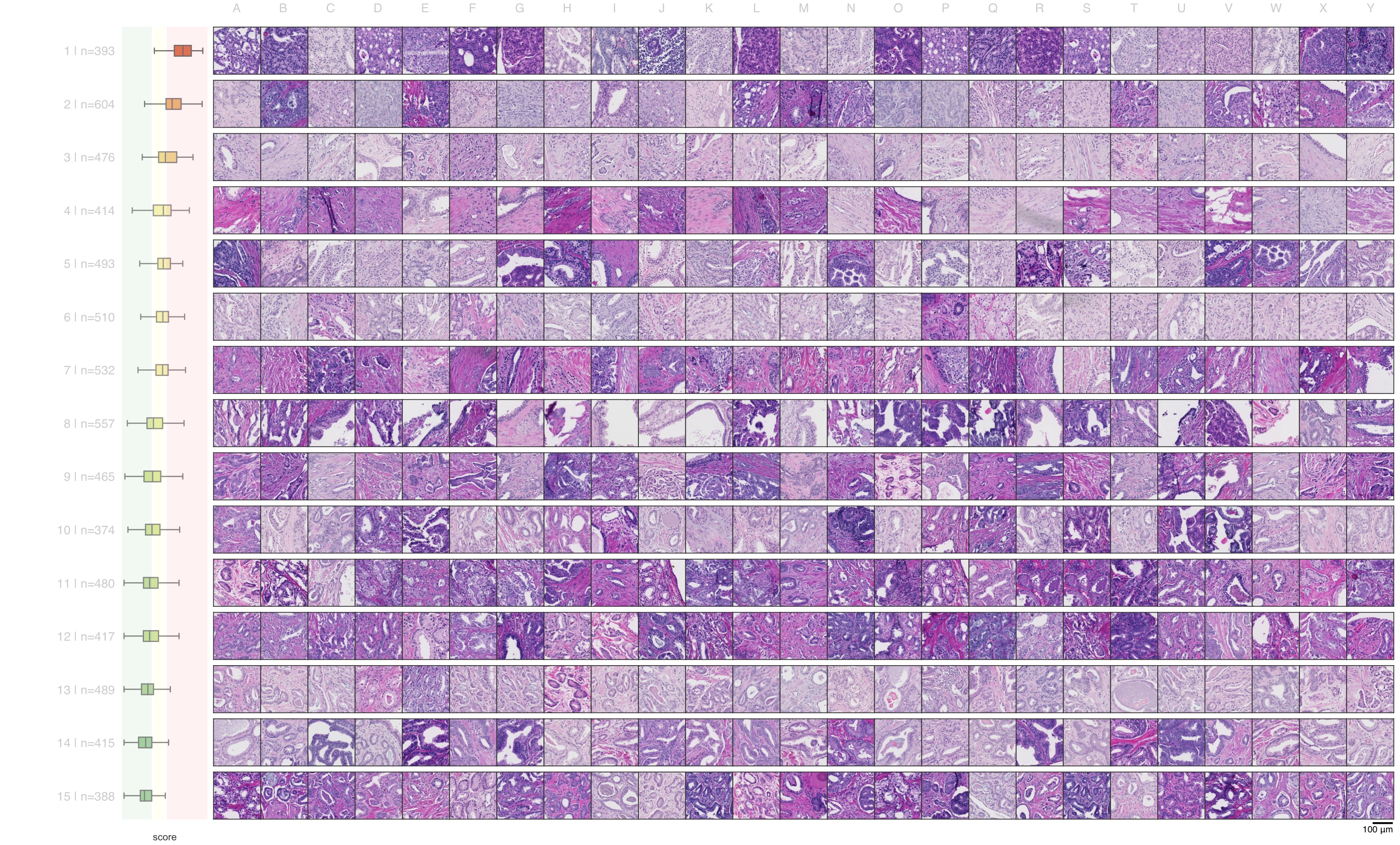
Supplementary Notes 2**

**Supplementary Figure 1:** 25 random examples per concept determined by Automatic Concept Explanations.

Sorted by the prediction of the DLS biomarker on the total TMA core. Best viewed digitally at high magnification.

The boxes shows the quartiles of the concept predictions while the whiskers extend to show the rest of the distribution,

except for outlier points that lie below the 25% or above 75% of the data, by 1.5 times the interquartile range.

|  |  |  | **Gleason pattern** | | |
| --- | --- | --- | --- | --- | --- |
| **Cluster** | **No tumor** | **Unclear pattern** | **3 / 3*** | **4 / 4*** | **5** |
| **0** | 0 | 0 | 1 | 24 | 0 |
| **1** | 0 | 0 | 4 | 20 | 1 |
| **2** | 4 | 0 | 12 | 6 | 3 |
| **3** | 10 | 1 | 9 | 3 | 2 |
| **4** | 2 | 1 | 7 | 15 | 0 |
| **5** | 0 | 0 | 12 | 13 | 0 |
| **6** | 2 | 0 | 16 | 6 | 1 |
| **7** | 4 | 4 | 11 | 6 | 0 |
| **8** | 0 | 0 | 22 | 3 | 0 |
| **9** | 0 | 2 | 19 | 4 | 0 |
| **10** | 0 | 0 | 21 | 4 | 0 |
| **11** | 0 | 0 | 20 | 5 | 0 |
| **12** | 1 | 0 | 24 | 0 | 0 |
| **13** | 0 | 2 | 18 | 5 | 0 |
| **14** | 0 | 0 | 24 | 1 | 0 |

**Supplemental Table 2:** Summary table of the 25 random examples of Supplemental Figure 1, assessed by JvI. The concepts predicted to have rapid biochemical recurrence (sorted at the top) show higher Gleason patterns compared to the lower sorted concepts. Due to the small tumor regions in the examples the Gleason pattern was not always readily recognizable. 3* means mainly 3 with possible component of 4. 4* means mainly 4 with possible component of 5


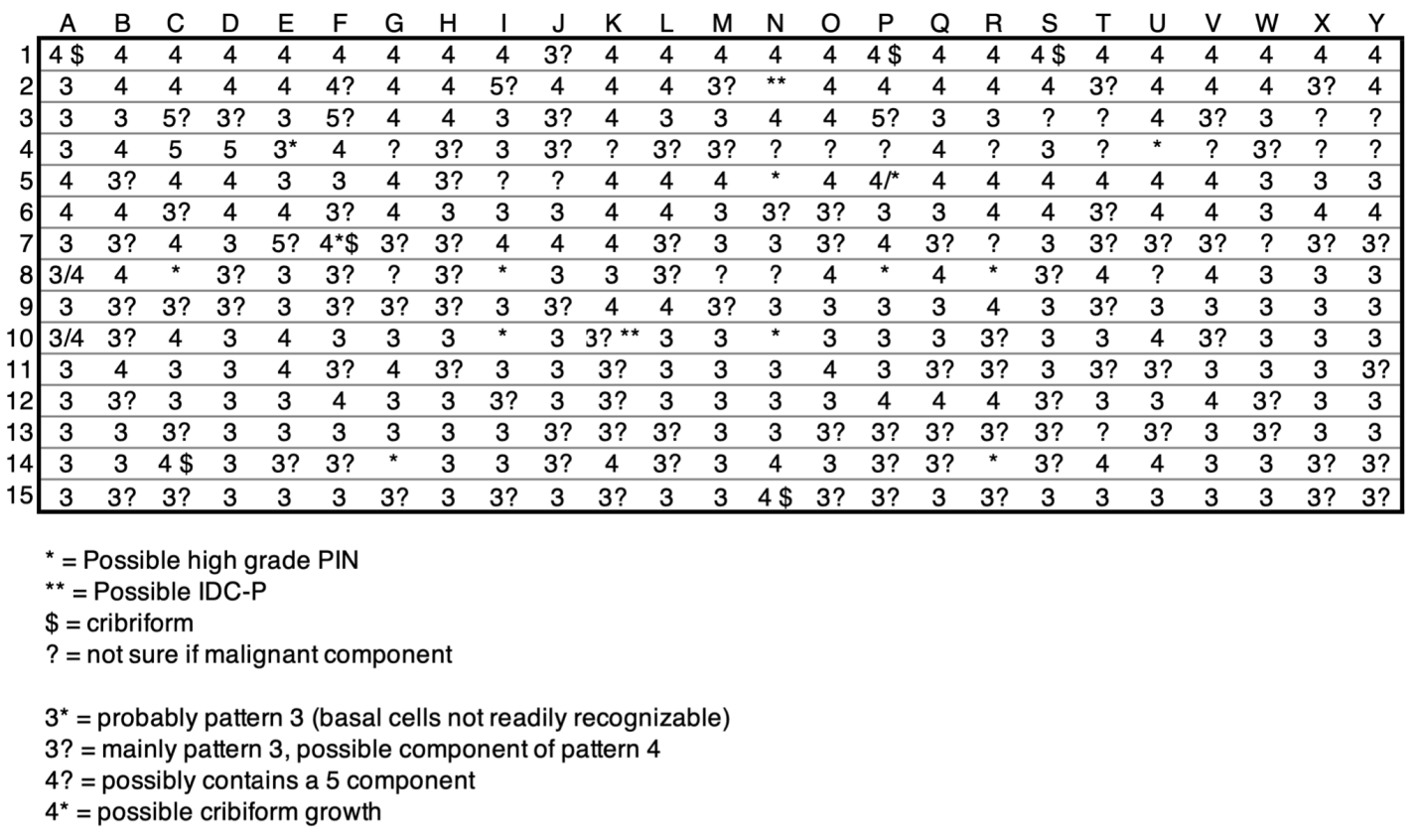


**Supplementary Table 3:** All 25 random examples of Supplemental Figure 1, as assessed by JvI.

**Supplementary References**

[1] [He T, Zhang Z, Zhang H, Zhang Z, Xie J, Li M. Bag of tricks for image classification with convolutional neural networks. Proceedings of the IEEE/CVF Conference on Computer Vision and Pattern Recognition, 2019, p. 558–67.](http://paperpile.com/b/4oaJCk/0C4uw)

[2] [Wightman R. PyTorch Image Models. GitHub; 2021. https://doi.org/](http://paperpile.com/b/4oaJCk/R8rzj)[10.5281/ZENODO.4414861](http://dx.doi.org/10.5281/ZENODO.4414861)[.](http://paperpile.com/b/4oaJCk/R8rzj)

[3] [DeVries T, Taylor GW. Improved Regularization of Convolutional Neural Networks with Cutout. arXiv [csCV] 2017.](http://paperpile.com/b/4oaJCk/pupvV)

[4] [Buslaev A, Iglovikov VI, Khvedchenya E, Parinov A, Druzhinin M, Kalinin AA. Albumentations: Fast and Flexible Image Augmentations. Information 2020;11:125.](http://paperpile.com/b/4oaJCk/Wwhua)

[5] [Howard J, Gugger S. Fastai: A Layered API for Deep Learning. Information 2020;11:108.](http://paperpile.com/b/4oaJCk/eqdzO)

[6] [Paszke A, Gross S, Massa F, Lerer A, Bradbury J, Chanan G, et al. PyTorch: An Imperative Style, High-Performance Deep Learning Library. arXiv [csLG] 2019.](http://paperpile.com/b/4oaJCk/ZASvN)

[7] [Zhang MR, Lucas J, Hinton G, Ba J. Lookahead Optimizer: k steps forward, 1 step back. arXiv [csLG] 2019.](http://paperpile.com/b/4oaJCk/9wkWS)

[8] [Liu L, Jiang H, He P, Chen W, Liu X, Gao J, et al. On the Variance of the Adaptive Learning Rate and Beyond. arXiv [csLG] 2019.](http://paperpile.com/b/4oaJCk/uGUEw)

[9] [Tan M, Le Q. Efficientnet: Rethinking model scaling for convolutional neural networks. International Conference on Machine Learning, PMLR; 2019, p. 6105–14.](http://paperpile.com/b/4oaJCk/ZG7CK)
