# Supplementary material for: Predicting biochemical recurrence of prostate cancer with artificial intelligence
Source: Commun Med (Lond). 2022 Jun 8;2:64. doi: 10.1038/s43856-022-00126-3 (PMC9177591; doi:10.1038/s43856-022-00126-3)
Supplement: Supplementary file 7 — Reporting Summary [file 43856_2022_126_MOESM7_ESM.pdf]

nature research

Corresponding author(s): Hans Pinckaers

Last updated by author(s): Apr 23, 2022

Reporting Summary

Nature Research wishes to improve the reproducibility of the work that we publish. This form provides structure for consistency and transparency in reporting. For further information on Nature Research policies, see our Editorial Policies and the Editorial Policy Checklist.

Statistics

For all statistical analyses, confirm that the following items are present in the figure legend, table legend, main text, or Methods section.

n/a	Confirmed	
☐	☒	The exact sample size (*n*) for each experimental group/condition, given as a discrete number and unit of measurement
☐	☒	A statement on whether measurements were taken from distinct samples or whether the same sample was measured repeatedly
☐	☒	The statistical test(s) used AND whether they are one- or two-sided<br>*Only common tests should be described solely by name; describe more complex techniques in the Methods section.*
☐	☒	A description of all covariates tested
☐	☒	A description of any assumptions or corrections, such as tests of normality and adjustment for multiple comparisons
☐	☒	A full description of the statistical parameters including central tendency (e.g. means) or other basic estimates (e.g. regression coefficient) AND variation (e.g. standard deviation) or associated estimates of uncertainty (e.g. confidence intervals)
☐	☒	For null hypothesis testing, the test statistic (e.g. *F*, *t*, *r*) with confidence intervals, effect sizes, degrees of freedom and *P* value noted<br>*Give P values as exact values whenever suitable.*
☒	☐	For Bayesian analysis, information on the choice of priors and Markov chain Monte Carlo settings
☒	☐	For hierarchical and complex designs, identification of the appropriate level for tests and full reporting of outcomes
☒	☐	Estimates of effect sizes (e.g. Cohen's *d*, Pearson's *r*), indicating how they were calculated

Our web collection on statistics for biologists contains articles on many of the points above.

Software and code

Policy information about availability of computer code

Data collection	N/A
Data analysis	Source code is available on https://zenodo.org/record/6480481

For manuscripts utilizing custom algorithms or software that are central to the research but not yet described in published literature, software must be made available to editors and reviewers. We strongly encourage code deposition in a community repository (e.g. GitHub). See the Nature Research guidelines for submitting code & software for further information.

Data

Policy information about availability of data

All manuscripts must include a data availability statement. This statement should provide the following information, where applicable:

- Accession codes, unique identifiers, or web links for publicly available datasets
- A list of figures that have associated raw data
- A description of any restrictions on data availability

The data can be requested from Prostate Cancer Biorepository Network (PCBN) via https://prostatebiorepository.org as mentioned in the paper.

Field-specific reporting

Please select the one below that is the best fit for your research. If you are not sure, read the appropriate sections before making your selection.

☒ Life sciences ☐ Behavioural & social sciences ☐ Ecological, evolutionary & environmental sciences

For a reference copy of the document with all sections, see nature.com/documents/nr-reporting-summary-flat.pdf

Life sciences study design

All studies must disclose on these points even when the disclosure is negative.

Sample size	No sample-size calculation were performed, we used available pre-existing datasets.
Data exclusions	We excluded patients if there was no or very little tumor visible in the tissue TMA cores, or if they did not have PSA nadir after surgery.
Replication	We trained a model on the tissue on different folds of the data. On every fold we did multiple runs, they gave similar results.
Randomization	We split the dataset into a training and test set based on the covariates on which the cohort was matched. So that the overall distribution would stay equal between the sets.
Blinding	Blinding was not relevant since we did not do any interventions. The sets were separated to investigate generalization of the computer algorithm to unseen data. The splitting was done using a random seed.

Reporting for specific materials, systems and methods

We require information from authors about some types of materials, experimental systems and methods used in many studies. Here, indicate whether each material, system or method listed is relevant to your study. If you are not sure if a list item applies to your research, read the appropriate section before selecting a response.

Materials & experimental systems

n/a	Involved in the study	
☒	☐	Antibodies
☒	☐	Eukaryotic cell lines
☒	☐	Palaeontology and archaeology
☒	☐	Animals and other organisms
☒	☐	Human research participants
☐	☒	Clinical data
☒	☐	Dual use research of concern

Methods

n/a	Involved in the study	
☒	☐	ChIP-seq
☒	☐	Flow cytometry
☒	☐	MRI-based neuroimaging

Clinical data

Policy information about clinical studies

All manuscripts should comply with the ICMJE guidelines for publication of clinical research and a completed CONSORT checklist must be included with all submissions.

Clinical trial registration	We are using pre-existing data collected in other research under institutional review board approval of their respective hospitals.
Study protocol	This is not an interventional study, as such we do not have a protocol.
Data collection	Data was collected at either the Johns Hopkins Hospital in Baltimore or New York Langone Medical Center, between 1993 and 2003.
Outcomes	Primary outcome was biochemical recurrence, secondary outcome of this study clinical recurrence of prostate cancer. To reiterate, this study only uses the data gathered in these mentioned source studies for machine learning. We did not perform any interventions on the participants. We received approval of both data source institutions for the current manuscript.
